# Supplementary material for: Modeling tissue-specific Drosophila metabolism identifies high sugar diet-induced metabolic dysregulation in muscle at reaction and pathway levels
Source: Nat Commun. 2026 Jan 19;17:1692. doi: 10.1038/s41467-026-68395-3 (PMC12910071; doi:10.1038/s41467-026-68395-3)
Supplement: Supplementary file 1 — Supplementary Information [file 41467_2026_68395_MOESM1_ESM.pdf]

Modeling tissue-specific *Drosophila* metabolism identifies high sugar diet-induced metabolic dysregulation in muscle at reaction and pathway levels

Sun Jin Moon[1*], Yanhui Hu[1], Monika Dzieciatkowska[2], Ah-Ram Kim[1], John M. Asara[3,4], Angelo D'Alessandro[2], Norbert Perrimon[1,5*]

[*]Correspondence: Sun Jin Moon and Norbert Perrimon

Emails: sunjin_moon@hms.harvard.edu and perrimon@genetics.med.harvard.edu

This document includes:

Supplementary Figures 2

Supplementary Figure 1 2

Supplementary Figure 2 4

Supplementary Figure 3 6

Supplementary Figure 4 8

Supplementary Figure 5 10

Supplementary Figure 6 12

Supplementary notes 14

Supplementary note 1: Pathway overlap between tissue-specific GEMs and experimental metabolomics 14

Supplementary note 2: Flux map representing central carbon metabolism 15

Supplementary note 3: Flux comparisons among FBA, FVA-sampling, and pFBA 16

3a. Total flux evaluation: 16

3b. NAD(P)-dependent internal cycling reactions evaluation: 16

3c. Flux boundary effects in FVA-sampling: 17

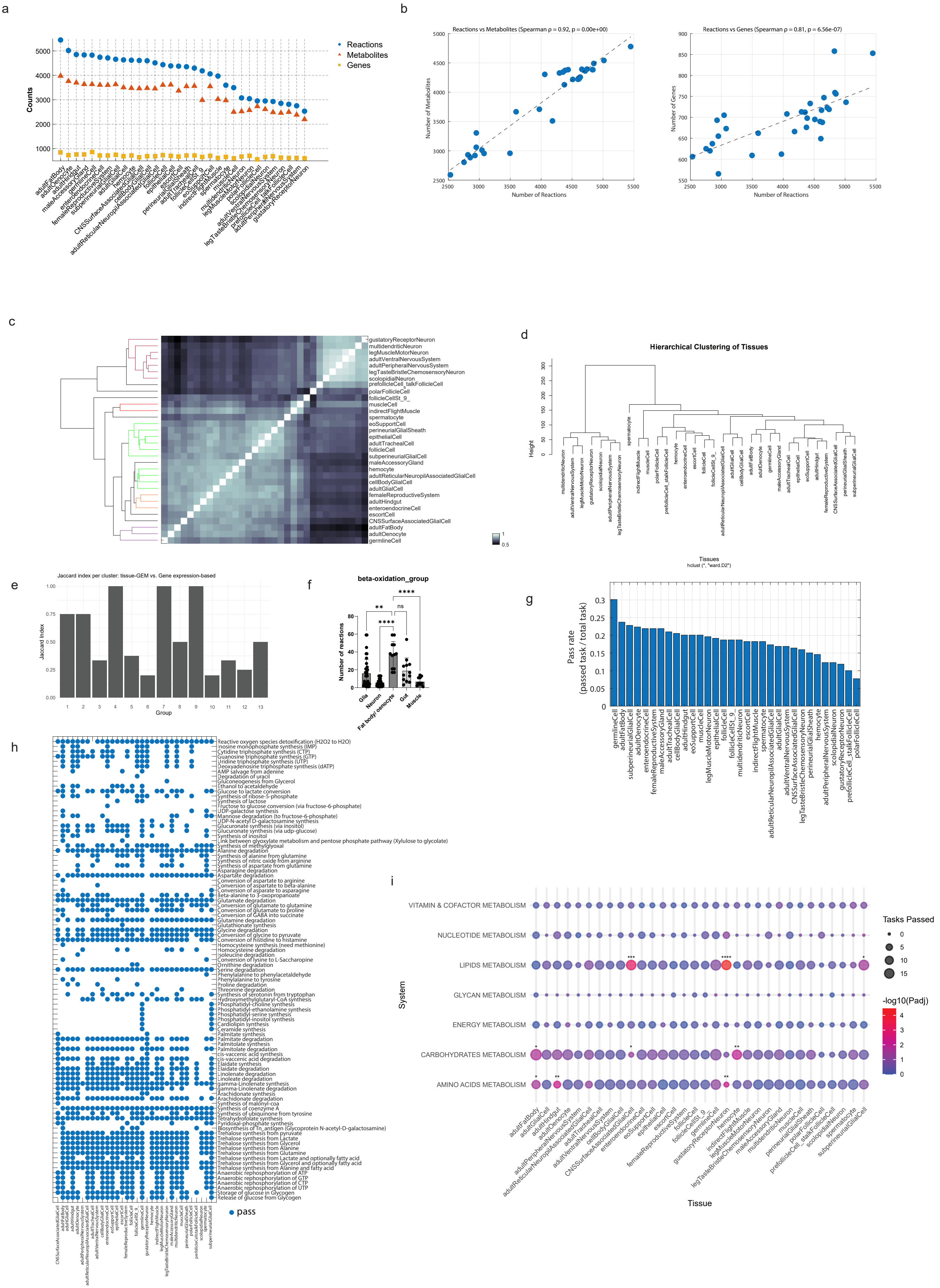

a
Counts
Reactions
Metabolites
Genes
b
Reactions vs Metabolites (Spearman ρ = 0.92, p = 0.00e+00)
Number of Metabolites
Number of Reactions
Reactions vs Genes (Spearman ρ = 0.81, p = 6.56e-07)
Number of Genes
Number of Reactions
c
d
Hierarchical Clustering of Tissues
Height
Tissues
hclust (*, "ward.D2")
e
Jaccard index per cluster: tissue-GEM vs. Gene expression-based
Jaccard Index
Group
f
beta-oxidation_group
Number of reactions
Glia
Neuron
Fat body/ oenocyte
Gut
Muscle
g
Pass rate
(passed task / total task)
h
pass
i
VITAMIN & COFACTOR METABOLISM
NUCLEOTIDE METABOLISM
LIPIDS METABOLISM
GLYCAN METABOLISM
ENERGY METABOLISM
CARBOHYDRATES METABOLISM
AMINO ACIDS METABOLISM
System
Tissue
Tasks Passed
-log10(Padj)

Supplementary Figure 1. Reconstruction of 32 tissue-specific genome-scale metabolic models (GEMs) in *Drosophila melanogaster*

a Dot plot showing the number of reactions, metabolites, and genes across tissue-specific GEMs. **b** Scatter plot of reactions vs. metabolites (left) and vs. genes (right) with spearman correlation and p-value. The Spearman correlation coefficient and the p-values are displayed. **c** The heatmap representing the pairwise similarity matrix of tissue-specific GEMs, with branch colors highlighting distinct clusters for major tissues, including neuron, glia, muscle, gut, and fat body. The color scale on the bottom right indicates the degree of similarity, where lighter colors represent higher similarity and darker colors indicate lower similarity. **d** Dendrogram of hierarchical clustering of tissues based on pseudo-bulk snRNA-seq data. Euclidean distance metric and Ward's method were used to group tissues with similar gene expression profiles. **e** The bar plot shows the Jaccard indices for each cluster, comparing tissue-GEM-based clusters with gene expression derived clusters. Each bar is reference to a group obtained from tissue-specific GEMs. Representative groups are group 1(fat body, oenocyte), group 3 (hindgut and enteroendocrine cell), group 7 (muscle and indirect muscle), group 4 (seven types of neurons), group 2, 5, and 11 (six types of glia). The rest clusters consist of other tissues. **f** Number of reactions in beta-oxidation fatty acid metabolism subsystem. Statistical significance was assessed using a Kruskal–Wallis test followed by Dunn's multiple comparisons test (5 groups; $p < 0.0001$; $n_{glia} = 36$, $n_{neuron} = 42$, $n_{fatbody/oenocyte} = 12$, $n_{gut} = 12$, $n_{muscle} = 12$). Data are shown as mean ± SD from biological replicates (n = 5). The significance was represented as follows: $* P < 0.05$, $** P < 0.01$, $*** P < 0.001$,$**** P < 0.0001$. **g** Metabolic task pass rate. Total number of metabolic tasks were divided by the total number of metabolic tasks. **h** Visualization of tissue-specific GEMs' ability to complete distinct metabolic tasks. Of 219 metabolic tasks, at least one tissue-GEM was able to complete 93 metabolic tasks as shown in the plot. Blue dots represent the models' passing the metabolic tasks. The presence of a dot indicates that the tissue-GEM was able to complete the respective task. Data can be found in supplementary data 1. **i** Tissue-specific enrichment of metabolic tasks across tissue-specific GEMs. Enrichment of metabolic tasks was assessed using Fisher's exact test applied to a 2×2 contingency table for each metabolic system–tissue pair. P values were corrected for multiple testing using the Benjamini–Hochberg FDR method. Each bubble represents a metabolic system–task association, where bubble size indicates the number of tasks passed in that system for the given tissue-specific GEM, and color intensity reflects the statistical significance. $* P < 0.05$, $** P < 0.01$, $*** P < 0.001$,$**** P < 0.0001$.

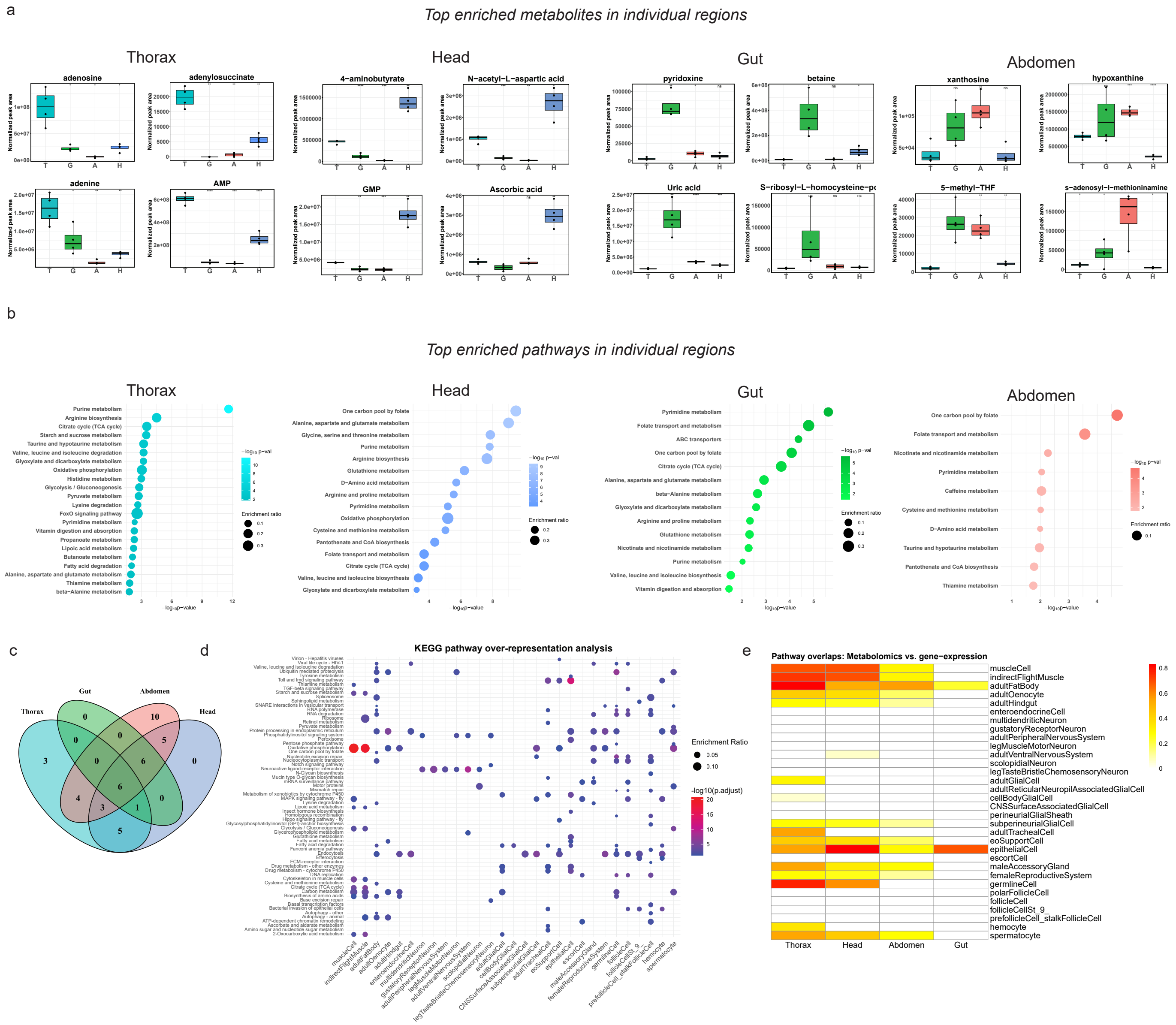

a
Top enriched metabolites in individual regions
Thorax
Head
Gut
Abdomen
adenosine
adenylosuccinate
4−aminobutyrate
N−acetyl−L−aspartic acid
pyridoxine
betaine
xanthosine
hypoxanthine
adenine
AMP
GMP
Ascorbic acid
Uric acid
S−ribosyl−L−homocysteine
5−methyl−THF
s−adenosyl−l−methioninamine
Normalized peak area
T
G
A
H
b
Top enriched pathways in individual regions
Thorax
Purine metabolism
Arginine biosynthesis
Citrate cycle (TCA cycle)
Starch and sucrose metabolism
Taurine and hypotaurine metabolism
Valine, leucine and isoleucine degradation
Glyoxylate and dicarboxylate metabolism
Oxidative phosphorylation
Histidine metabolism
Glycolysis / Gluconeogenesis
Pyruvate metabolism
Lysine degradation
FoxO signaling pathway
Pyrimidine metabolism
Vitamin digestion and absorption
Propanoate metabolism
Lipoic acid metabolism
Butanoate metabolism
Fatty acid degradation
Alanine, aspartate and glutamate metabolism
Thiamine metabolism
beta−Alanine metabolism
Head
One carbon pool by folate
Alanine, aspartate and glutamate metabolism
Glycine, serine and threonine metabolism
Purine metabolism
Arginine biosynthesis
Glutathione metabolism
D−Amino acid metabolism
Arginine and proline metabolism
Pyrimidine metabolism
Oxidative phosphorylation
Cysteine and methionine metabolism
Pantothenate and CoA biosynthesis
Folate transport and metabolism
Citrate cycle (TCA cycle)
Valine, leucine and isoleucine biosynthesis
Glyoxylate and dicarboxylate metabolism
Gut
Pyrimidine metabolism
Folate transport and metabolism
ABC transporters
One carbon pool by folate
Citrate cycle (TCA cycle)
Alanine, aspartate and glutamate metabolism
beta−Alanine metabolism
Glyoxylate and dicarboxylate metabolism
Arginine and proline metabolism
Glutathione metabolism
Nicotinate and nicotinamide metabolism
Purine metabolism
Valine, leucine and isoleucine biosynthesis
Vitamin digestion and absorption
Abdomen
One carbon pool by folate
Folate transport and metabolism
Nicotinate and nicotinamide metabolism
Pyrimidine metabolism
Caffeine metabolism
Cysteine and methionine metabolism
D−Amino acid metabolism
Taurine and hypotaurine metabolism
Pantothenate and CoA biosynthesis
Thiamine metabolism
−log10 p−val
Enrichment ratio
−log10p−value
c
Thorax
Gut
Abdomen
Head
d
KEGG pathway over-representation analysis
Enrichment Ratio
-log10(p.adjust)
e
Pathway overlaps: Metabolomics vs. gene−expression
muscleCell
indirectFlightMuscle
adultFatBody
adultOenocyte
adultHindgut
enteroendocrineCell
multidendriticNeuron
gustatoryReceptorNeuron
adultPeripheralNervousSystem
legMuscleMotorNeuron
adultVentralNervousSystem
scolopidialNeuron
legTasteBristleChemosensoryNeuron
adultGlialCell
adultReticularNeuropilAssociatedGlialCell
cellBodyGlialCell
CNSSurfaceAssociatedGlialCell
perineurialGlialSheath
subperineurialGlialCell
adultTrachealCell
eoSupportCell
epithelialCell
escortCell
maleAccessoryGland
femaleReproductiveSystem
germlineCell
polarFollicleCell
follicleCell
follicleCellSt_9_
prefollicleCell_stalkFollicleCell
hemocyte
spermatocyte
Thorax
Head
Abdomen
Gut

Supplementary Figure 2. Validation of tissue-specific GEMs through regional metabolomics and pathway analysis

a Boxplots represent the normalized peak area of selected metabolites in thorax (T), head (H), gut (G), and abdomen (A). Center line is median; box limits are first and third quartiles; whiskers are 1.5 × interquartile range (n=4). One-way ANOVA was used to calculate p-values across groups. Pairwise comparisons between each tissue and thorax were then conducted using post-hoc t-tests with Benjamini–Hochberg (BH) correction for multiple comparisons. $* \, P < 0.05$, $** \, P < 0.01$, $*** \, P < 0.001$, $**** \, P < 0.0001$. **b** Bubble plots show significantly enriched metabolic pathways across four dissected *Drosophila* regions: thorax, head, gut, and abdomen. Each bubble represents a KEGG pathway. The x-axis displays the enrichment significance as $-\log_{10}$(p-value), calculated using a Fisher's exact over-representation test. Bubble size corresponds to the enrichment ratio (observed/expected). Bubble color reflects region-specific plots (cyan: thorax, blue: head, green: gut, pink: abdomen). **c** Venn diagram of enriched pathways across regions. **d** KEGG enrichment analysis (over-representation) for pseudo-bulk snRNA-seq data. Each dot represents a KEGG pathway enriched in a specific tissue or cell type, based on over-representation analysis of genes from pseudo-bulk single-nucleus RNA-seq (snRNA-seq) data. The x-axis shows *Drosophila* tissues/cell types; the y-axis lists KEGG pathways. Dot color represents the statistical significance of enrichment, scaled as $-\log_{10}$(adjusted p-value), with red colors indicating higher significance. Dot size reflects the enrichment ratio (observed vs. expected counts). Enrichment was assessed using a Fisher's exact test with Benjamini–Hochberg correction. Tissues with no significantly enriched pathways in a given category are left blank. **e** Pathway overlaps between metabolomics and gene-expression analysis. The heatmap shows the weighted Jaccard index between pathways enriched from metabolomics (per dissected region: thorax, head, abdomen, gut) and pathways inferred from KEGG over-representation analysis based on gene expression levels of individual tissues. Rows represent *Drosophila* cell types or tissues, and columns correspond to the four anatomical regions profiled in metabolomics. Color intensity reflects the degree of overlap, with red indicating stronger pathway agreement (weighted Jaccard index, scale 0–0.8). Blank cells indicate no overlap or missing data.

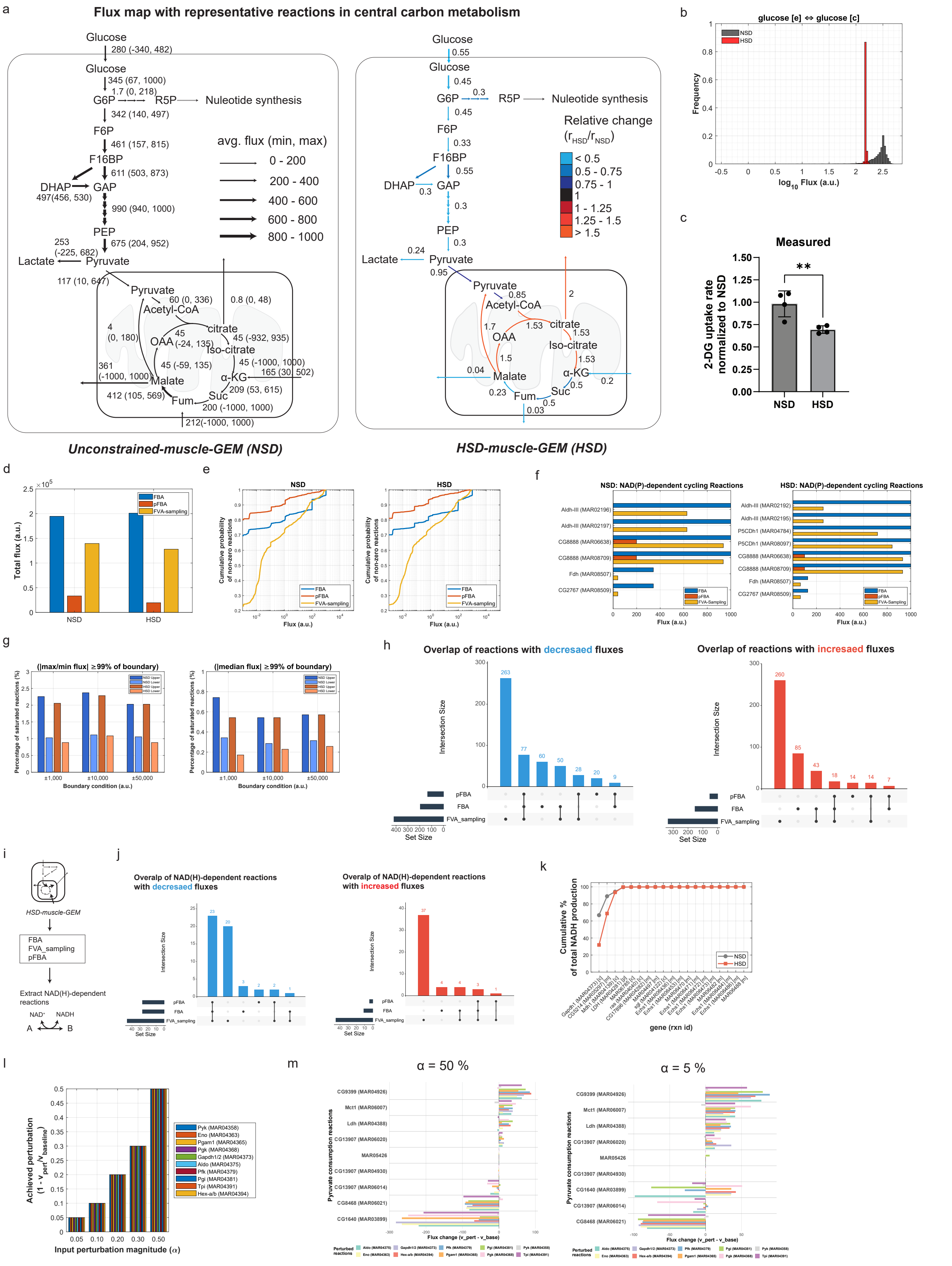

a
Flux map with representative reactions in central carbon metabolism
Glucose
280 (-340, 482)
Glucose
345 (67, 1000)
1.7 (0, 218)
G6P
R5P
Nuleotide synthesis
342 (140, 497)
F6P
461 (157, 815)
F16BP
611 (503, 873)
DHAP
GAP
497(456, 530)
990 (940, 1000)
PEP
675 (204, 952)
253
(-225, 682)
Lactate
Pyruvate
117 (10, 647)
Pyruvate
60 (0, 336)
0.8 (0, 48)
Acetyl-CoA
citrate
OAA
Iso-citrate
Malate
α-KG
Fum
Suc
avg. flux (min, max)
0 - 200
200 - 400
400 - 600
600 - 800
800 - 1000
Unconstrained-muscle-GEM (NSD)
HSD-muscle-GEM (HSD)
Relative change
(r_{HSD}/r_{NSD})
< 0.5
0.5 - 0.75
0.75 - 1
1
1 - 1.25
1.25 - 1.5
> 1.5
b
glucose [e] ⇔ glucose [c]
NSD
HSD
Frequency
log_{10} Flux (a.u.)
c
Measured
2-DG uptake rate normalized to NSD
NSD
HSD
d
Total flux (a.u.)
FBA
pFBA
FVA-sampling
NSD
HSD
e
NSD
HSD
Cumulative probability of non-zero reactions
Flux (a.u.)
f
NSD: NAD(P)-dependent cycling Reactions
HSD: NAD(P)-dependent cycling Reactions
Aldh-III (MAR02196)
Aldh-III (MAR02197)
CG8888 (MAR06638)
CG8888 (MAR08709)
Fdh (MAR08507)
CG2767 (MAR08509)
Aldh-III (MAR02192)
Aldh-III (MAR02195)
P5CDh1 (MAR04784)
P5CDh1 (MAR08097)
g
(|max/min flux| ≥99% of boundary)
(|median flux| ≥99% of boundary)
Percentage of saturated reactions (%)
Boundary condition (a.u.)
h
Overlap of reactions with decresaed fluxes
Overlap of reactions with incresaed fluxes
Intersection Size
Set Size
pFBA
FBA
FVA_sampling
i
HSD-muscle-GEM
FBA
FVA_sampling
pFBA
Extract NAD(H)-dependent reactions
NAD+
NADH
A
B
j
Overalp of NAD(H)-dependent reactions with decresaed fluxes
Overalp of NAD(H)-dependent reactions with increased fluxes
k
Cumulative % of total NADH production
gene (rxn id)
NSD
HSD
l
Achieved perturbation ($1 - v_{pert}/v_{baseline}$)
Input perturbation magnitude (α)
Pyk (MAR04358)
Eno (MAR04363)
Pgam1 (MAR04365)
Pgk (MAR04368)
Gapdh1/2 (MAR04373)
Aldo (MAR04375)
Pfk (MAR04379)
Pgi (MAR04381)
Tpi (MAR04391)
Hex-a/b (MAR04394)
m
α = 50 %
α = 5 %
Pyruvate consumption reactions
Flux change (v_pert - v_base)
Perturbed reactions

Supplementary Figure 3. Constraint-based flux analyses predict perturbations in NAD(H)-dependent reactions in muscle under high sugar diet

a Constraint-based flux analysis shows representative fluxes in central carbon metabolism. Arrows indicate the direction and magnitude of average predicted fluxes (arbitrary units), with the corresponding minimum and maximum fluxes for each reaction shown in parentheses. Representative fluxes were obtained from FVA-sampling. Only key reactions in central pathways are shown, while detailed reaction sets are provided in Supplementary Data 3c–d. **b** Model-predicted flux distribution of glucose uptake reaction (glucose[e] ⇔ glucose[c]) (n_{sim}=10,000). **c** Glucose uptake in thoracic muscle. 2-deoxyglucose (2-DG) uptake rate was measured in dissected thoracic muscles from adult male flies fed NSD or HSD after 5 days using the kit (Promega: J1341). Data are shown as mean ± SD from biological replicates (n = 5). Statistical significance was assessed using a two-tailed unpaired t-test; ** $p < 0.01$. **d** Total flux in the muscle-GEM was quantified. **e** Empirical cumulative distribution functions of non-zero reaction fluxes. **f** NAD(P)-dependent cycling reactions. Horizontal bars show flux magnitudes from FBA, pFBA, and FVA_sampling analysis for the top cycling reactions ranked by flux reduction. **g** Percentage of reactions exhibiting saturation across boundary conditions. **h** Overlap of reactions with commonly decreased (blue) and increased (red) fluxes under HSD compared to NSD, as identified by pFBA, FBA, and FVA sampling analyses. UpSet plots were used to indicate total and intersecting sets of reactions showing consistent flux changes across methods. **i** Workflow for identifying commonly perturbed NAD(H)-dependent reactions in HSD-muscle-GEM. Differential reaction fluxes were computed using FBA, FVA sampling, and pFBA. **j** Overlap of NAD(H)-dependent reactions. **k** Cumulative percentage contribution of individual NADH-producing reactions to total NADH production capacity under NSD and HSD conditions. **l** Bar plots showing achieved perturbation magnitude. Each colored bar represents the achieved perturbation for a specific enzyme across conditions, with baseline flux estimated as the median from 5,000 FVA sampling points per reaction. **m** Bar plots showing changes in flux through individual pyruvate-consuming reactions following 50% (left) and 5% (right) flux reductions in each targeted glycolytic enzyme.

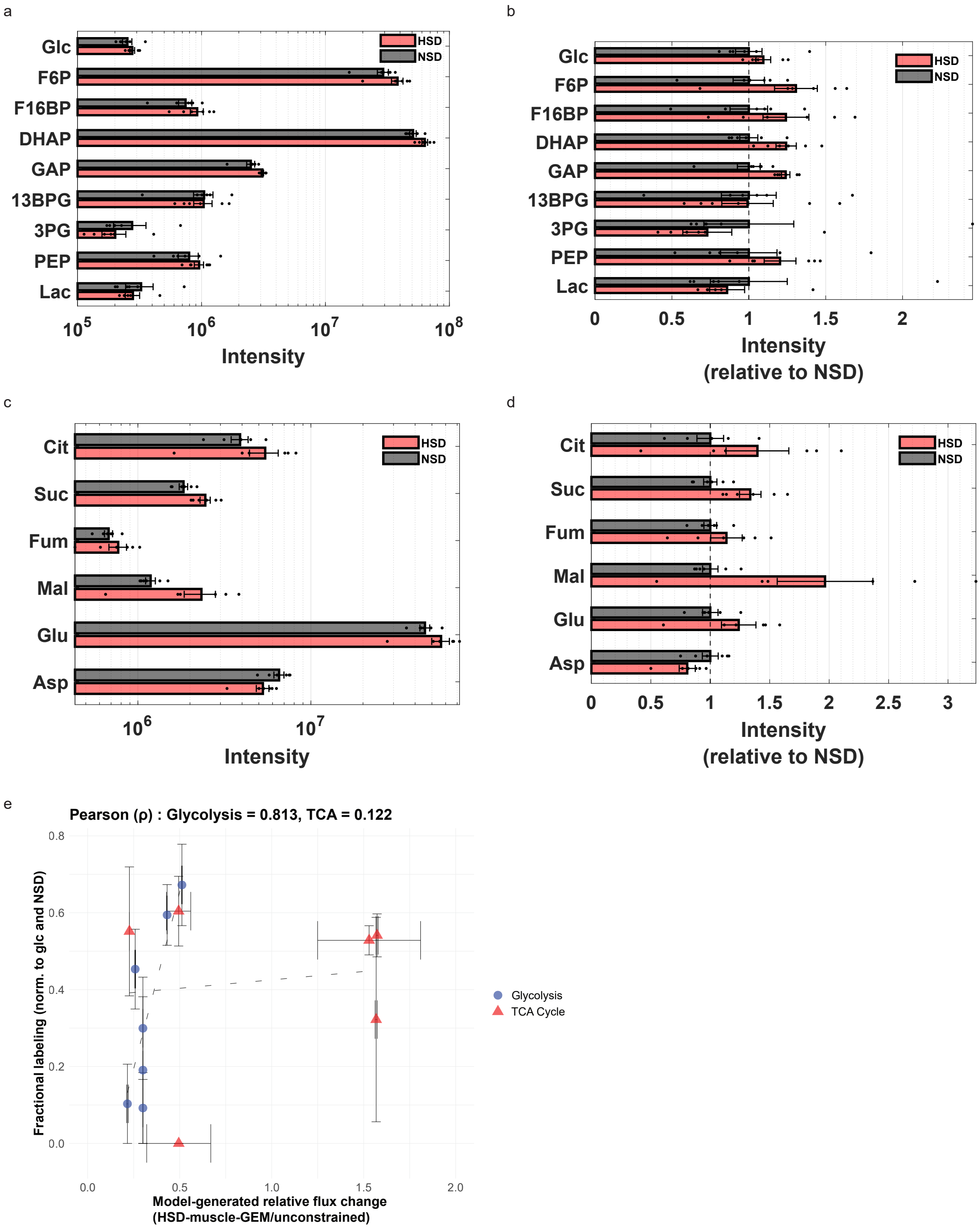

a
Glc
F6P
F16BP
DHAP
GAP
13BPG
3PG
PEP
Lac
HSD
NSD
10^5
10^6
10^7
10^8
Intensity
b
Glc
F6P
F16BP
DHAP
GAP
13BPG
3PG
PEP
Lac
HSD
NSD
0
0.5
1
1.5
2
Intensity
(relative to NSD)
c
Cit
Suc
Fum
Mal
Glu
Asp
HSD
NSD
10^6
10^7
Intensity
d
Cit
Suc
Fum
Mal
Glu
Asp
HSD
NSD
0
0.5
1
1.5
2
2.5
3
Intensity
(relative to NSD)
e
Pearson (ρ) : Glycolysis = 0.813, TCA = 0.122
Fractional labeling (norm. to glc and NSD)
0.0
0.2
0.4
0.6
0.8
0.0
0.5
1.0
1.5
2.0
Model-generated relative flux change
(HSD-muscle-GEM/unconstrained)
Glycolysis
TCA Cycle

Supplementary Figure 4. Model-predicted decreases in glycolytic flux, including GAPDH, validated through ^{13}C-glucose tracing

(a, b) absolute (**a**) and relative (**b**) intensities of glycolytic intermediates detected from LC/MS analysis in response to HSD and NSD. Bars represent mean ± SEM, and individual points indicate biological replicates (n = 6). M+x denotes a mass isotopomer containing x ^{13}C atoms. For (b), values are normalized to the mean NSD intensity for each metabolite. Measured metabolites: Glc (glucose), F6P (fructose-6-phosphate), F16BP (fructose-1,6-bisphosphate), DHAP (dihydroxyacetone phosphate), GAP (glyceraldehyde-3-phosphate), 13BPG (1,3-bisphosphoglycerate), 3PG (3-phosphoglycerate), PEP (phosphoenolpyruvate), and Lac (lactate). **(c, d)** Absolute (**c**) and relative (**d**) intensities of TCA cycle intermediates and amino acids. Metabolites: Cit (citrate), Suc (succinate), Fum (fumarate), Mal (malate), Glu (glutamate), Asp (aspartate). Bars represent mean ± SEM, and individual points indicate biological replicates (n = 6). M+x denotes a mass isotopomer containing x ^{13}C atoms. **e.** Scatter plot comparing experimentally measured fractional labeling (normalized to glucose and NSD) with relative flux changes predicted by the HSD muscle-GEM model for glycolytic (blue circles) and TCA cycle (red triangles) intermediates. Fractional labeling values (y-axis) are shown as mean ± SEM from biologically independent replicates (n = 6), and model-predicted flux values (x-axis) are shown as mean ± SEM from flux sampling simulations (n = 10,000). The dashed red line represents a linear regression fit. A two-sided Pearson's correlation coefficients (ρ) are shown for glycolysis and TCA cycle metabolites.

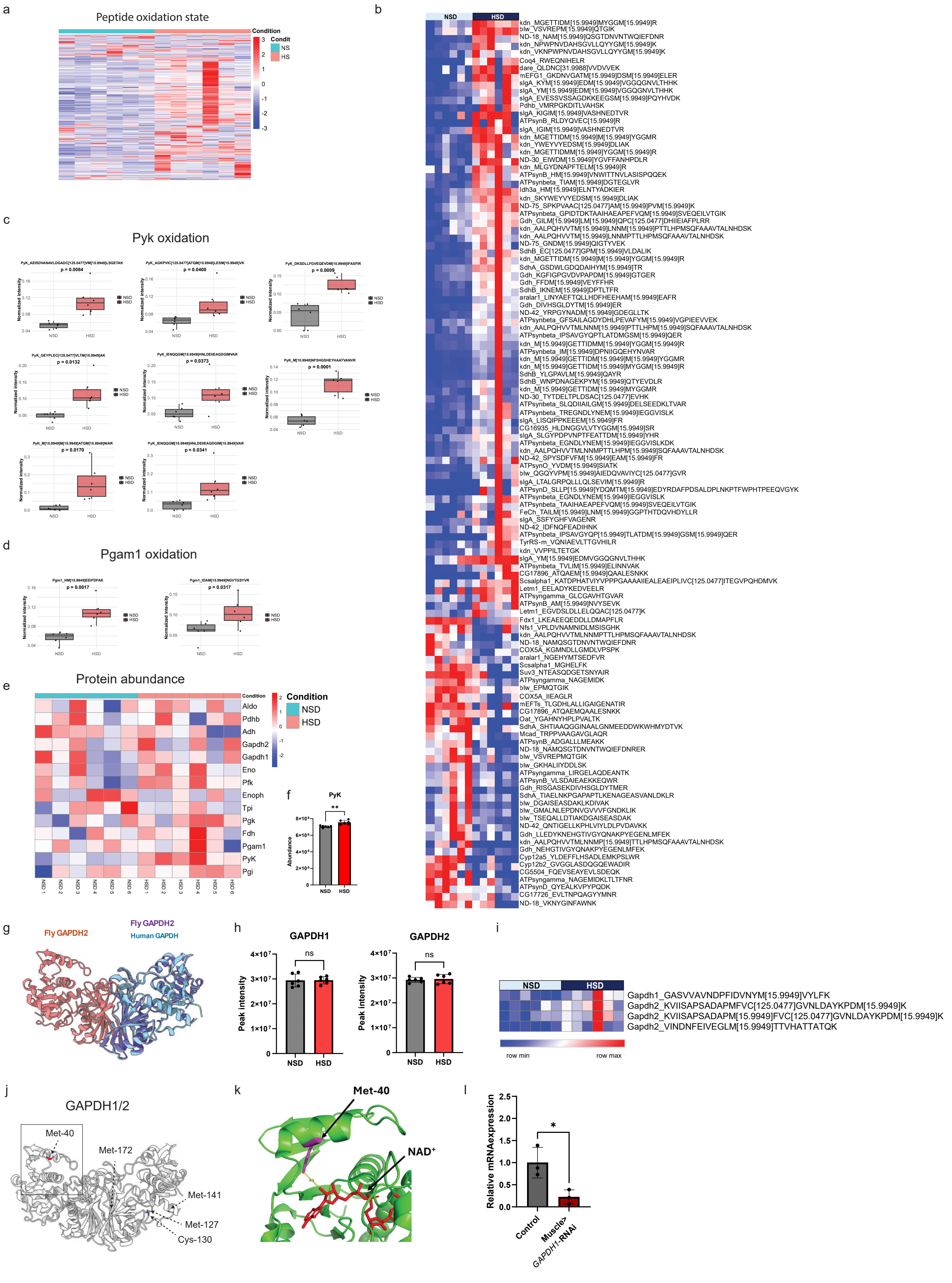

a
Peptide oxidation state
Condition
Condit
NS
HS
b
NSD
HSD
kdn_MGETTIDM[15.9949]MYGGM[15.9949]R
blw_VSVREPM[15.9949]QTGIK
ND-18_NAM[15.9949]QSGTDNVNTWQIEFDNR
kdn_NPWPNVDAHSGVLLQYYGM[15.9949]K
kdn_VKNPWPNVDAHSGVLLQYYGM[15.9949]K
Coq4_RWEQNIHELR
dare_QLDNC[31.9988]VVDVVEK
mEFG1_GKDNVGATM[15.9949]DSM[15.9949]ELER
sIgA_KYM[15.9949]EDM[15.9949]VGGQGNVLTHHK
sIgA_YM[15.9949]EDM[15.9949]VGGQGNVLTHHK
sIgA_EVESSVSSAGDKKEEGSM[15.9949]PQYHVDK
Pdhb_VMRPGKDITLVAHSK
sIgA_KIGIM[15.9949]VASHNEDTVR
ATPsynB_RLDYQVEC[15.9949]R
sIgA_IGIM[15.9949]VASHNEDTVR
kdn_MGETTIDM[15.9949]M[15.9949]YGGMR
kdn_YWEYVYEDSM[15.9949]DLIAK
kdn_MGETTIDMM[15.9949]YGGM[15.9949]R
ND-30_EIWDM[15.9949]YGVFFANHPDLR
kdn_MLGYDNAPFTELM[15.9949]R
ATPsynB_HM[15.9949]VNWITTNVLASISPQEK
ATPsynbeta_TIAM[15.9949]DGTEGLVR
Idh3a_HM[15.9949]ELNTYADKIER
kdn_SKYWEYVYEDSM[15.9949]DLIAK
ND-75_SPKPVAAC[125.0477]AM[15.9949]PVM[15.9949]K
ATPsynbeta_GPIDTDKTAAIHAEAPEFVQM[15.9949]SVEQEILVTGIK
Gdh_GILM[15.9949]LM[15.9949]QPC[125.0477]DHIIEIAFPLRR
kdn_AALPQHVVTM[15.9949]LNNM[15.9949]PTTLHPMSQFAAAVTALNHDSK
kdn_AALPQHVVTM[15.9949]LNNMPTTLHPMSQFAAAVTALNHDSK
ND-75_GNDM[15.9949]QIGTYVEK
SdhB_EC[125.0477]GPM[15.9949]VLDALIK
kdn_MGETTIDM[15.9949]M[15.9949]YGGM[15.9949]R
SdhA_GSDWLGDQDAIHYM[15.9949]TR
Gdh_KGFIGPGVDVPAPDM[15.9949]GTGER
Gdh_FFDM[15.9949]VEYFFHR
SdhB_IKNEM[15.9949]DPTLFR
aralar1_LINYAEFTQLLHDFHEEHAM[15.9949]EAFR
Gdh_DIVHSGLDYTM[15.9949]ER
ND-42_YRPGYNADM[15.9949]GDEGLLTK
ATPsynbeta_GFSAILAGDYDHLPEVAFYM[15.9949]VGPIEEVVEK
kdn_AALPQHVVTMLNNM[15.9949]PTTLHPM[15.9949]SQFAAAVTALNHDSK
ATPsynbeta_IPSAVGYQPTLATDMGSM[15.9949]QER
kdn_M[15.9949]GETTIDMM[15.9949]YGGM[15.9949]R
ATPsynbeta_IM[15.9949]DPNIIGQEHYNVAR
kdn_M[15.9949]GETTIDM[15.9949]M[15.9949]YGGMR
kdn_M[15.9949]GETTIDM[15.9949]MYGGM[15.9949]R
SdhB_YLGPAVLM[15.9949]QAYR
SdhB_WNPDNAGEKPYM[15.9949]QTYEVDLR
kdn_M[15.9949]GETTIDM[15.9949]MYGGMR
ND-30_TYTDELTPLDSAC[125.0477]EVHK
ATPsynbeta_SLQDIIAILGM[15.9949]DELSEEDKLTVAR
ATPsynbeta_TREGNDLYNEM[15.9949]IEGGVISLK
sIgA_LISQIPPKEEEM[15.9949]FR
CG16935_HLDNGGVLVTYGGM[15.9949]SR
sIgA_SLGYPDPVNPTFEATTDM[15.9949]YHR
ATPsynbeta_EGNDLYNEM[15.9949]IEGGVISLKDK
kdn_AALPQHVVTMLNNMPTTLHPM[15.9949]SQFAAAVTALNHDSK
ND-42_SPYSDFVFM[15.9949]EAM[15.9949]FR
ATPsynO_YVDM[15.9949]SIATK
blw_QGQYVPM[15.9949]AIEDQVAVIYC[125.0477]GVR
sIgA_LTALGRPQLLLQLSEVIM[15.9949]R
ATPsynD_SLLP[15.9949]YDQMTM[15.9949]EDYRDAFPDSALDPLNKPTFWPHTPEEQVGYK
ATPsynbeta_EGNDLYNEM[15.9949]IEGGVISLK
ATPsynbeta_TAAIHAEAPEFVQM[15.9949]SVEQEILVTGIK
FeCh_TAILM[15.9949]LNM[15.9949]GGPTHTDQVHDYLLR
sIgA_SSFYGHFVAGENR
ND-42_IDFNQFEADIHNK
ATPsynbeta_IPSAVGYQP[15.9949]TLATDM[15.9949]GSM[15.9949]QER
TyrRS-m_VQNIAEVLTTGVHILR
kdn_VVPPILTETGK
sIgA_YM[15.9949]EDMVGGQGNVLTHHK
ATPsynbeta_TVLIM[15.9949]ELINNVAK
CG17896_ATQAEM[15.9949]QAALESNKK
Scsalpha1_KATDPHATVIYVPPPGAAAAIIEALEAEIPLIVC[125.0477]ITEGVPQHDMVK
Letm1_EELADYKEDVEELR
ATPsyngamma_GLCGAVHTGVAR
ATPsynB_AM[15.9949]NVYSEVK
Letm1_EGVDSLDLLELQQAC[125.0477]K
Fdx1_LKEAEEQEDDLLDMAPFLR
Nfs1_VPLDVNAMNIDLMSISGHK
kdn_AALPQHVVTMLNNMPTTLHPMSQFAAAVTALNHDSK
ND-18_NAMQSGTDNVNTWQIEFDNR
COX5A_KGMNDLLGMDLVPSPK
aralar1_NGEHYMTSEDFVR
Scsalpha1_MGHELFK
Suv3_NTEASQDGETSNYAIR
ATPsyngamma_NAGEMIDK
blw_EPMQTGIK
COX5A_IIEAGLR
mEFTs_TLGDHLALLIGAIGENATIR
CG17896_ATQAEMQAALESNKK
Oat_YGAHNYHPLPVALTK
SdhA_SHTIAAQGGINAALGNMEEDDWKWHMYDTVK
Mcad_TRPPVAAGAVGLAQR
ATPsynB_ADGALLLMEAKK
ND-18_NAMQSGTDNVNTWQIEFDNRER
blw_VSVREPMQTGIK
blw_GKHALIIYDDLSK
ATPsyngamma_LIRGELAQDEANTK
ATPsynB_VLSDAIEAEKKEQWR
Gdh_RISGASEKDIVHSGLDYTMER
SdhA_TIAELNKPGAPAPTLKENAGEASVANLDKLR
blw_DGAISEASDAKLKDIVAK
blw_GMALNLEPDNVGVVVFGNDKLIK
blw_TSEQALLDTIAKDGAISEASDAK
ND-42_QNTIGELLKPHLVIYLDLPVDAVKK
Gdh_LLEDYKNEHGTIVGYQNAKPYEGENLMFEK
kdn_AALPQHVVTMLNNMP[15.9949]TTLHPMSQFAAAVTALNHDSK
Gdh_NEHGTIVGYQNAKPYEGENLMFEK
Cyp12a5_YLDEFFLHSADLEMKPSLWR
Cyp12b2_GVGGLASDQGQEWADIR
CG5504_FQEVSEAYEVLSDEQK
ATPsyngamma_NAGEMIDKLTLTFNR
ATPsynD_QYEALKVPYPQDK
CG17726_EVLTNPQAGYYMNR
ND-18_VKNYGINFAWNK
c
Pyk oxidation
Normalized intensity
NSD
HSD
p = 0.0084
p = 0.0400
p = 0.0009
p = 0.0132
p = 0.0373
p = 0.0001
p = 0.0170
p = 0.0341
d
Pgam1 oxidation
p = 0.0017
p = 0.0317
e
Protein abundance
Condition
NSD
HSD
Aldo
Pdhb
Adh
Gapdh2
Gapdh1
Eno
Pfk
Enoph
Tpi
Pgk
Fdh
Pgam1
PyK
Pgi
NSD 1
NSD 2
NSD 3
NSD 4
NSD 5
NSD 6
HSD 1
HSD 2
HSD 3
HSD 4
HSD 5
HSD 6
f
PyK
Abundance
**
NSD
HSD
g
Fly GAPDH2
Fly GAPDH2
Human GAPDH
h
GAPDH1
GAPDH2
Peak intensity
ns
NSD
HSD
i
NSD
HSD
Gapdh1_GASVVAVNDPFIDVNYM[15.9949]VYLFK
Gapdh2_KVIISAPSADAPMFVC[125.0477]GVNLDAYKPDM[15.9949]K
Gapdh2_KVIISAPSADAPM[15.9949]FVC[125.0477]GVNLDAYKPDM[15.9949]K
Gapdh2_VINDNFEIVEGLM[15.9949]TTVHATTATQK
row min
row max
j
GAPDH1/2
Met-40
Met-172
Met-141
Met-127
Cys-130
k
Met-40
NAD⁺
l
Relative mRNAexpression
*
Control
Muscle>
GAPDH1-RNAi

Supplementary Figure 5. Model-predicted decreases in glycolytic flux correlate with increased redox modification of glycolytic enzymes

a Heatmap showing the oxidation state values of significantly altered peptides under normal sugar diet (NSD) and high sugar diet (HSD) conditions (n=6). Each row represents a peptide; each column corresponds to a biological replicate. Color scale represents oxidation state, with red indicating greater oxidation. **b** Heatmap showing mitochondrial peptide oxidation states (n=6). The color scale reflects z-score normalized oxidation states, with red indicating increased oxidation levels and blue indicating decreased oxidation levels relative to mean of each row. The raw data is found in the supplementary data. 5a**. (c, d) (c)** Boxplot showing the normalized intensity of significantly oxidized peptides for pyruvate kinase (Pyk) and **(d)** phosphoglycerate mutase 1 (Pgam1) under NSD and HSD conditions**.** Center line is median; box limits are first and third quartiles; whiskers are 1.5 × interquartile range; points represent biologically independent replicates (n = 6). Statistical significance was assessed using a two-tailed unpaired t-test and the p-value is shown above the plot. **e** Heatmap of glycolytic enzyme abundances in muscle tissues under NSD and HSD conditions (n=6)**.** Rows correspond to individual enzymes and columns to biological replicates under each condition. **f** Bar plot showing relative abundance of pyruvate kinase (PyK) in NSD and HSD samples. Data are shown as mean ± SD from biological replicates (n = 6). Statistical significance was assessed using a two-tailed unpaired t-test; ** $p < 0.01$. g Predicted enzyme structures and the interactions among fly GAPDH1 (FBgn0001091), fly GAPDH2 (FBgn0001091) and the overlapped structure of human GAPDH by Alphafold2-multimer. **h** Bar plots show peak intensity values for GAPDH1 and GAPDH2 proteins measured in muscle under NSD and HSD conditions. Data are shown as mean ± SD from biological replicates (n = 6). Statistical significance was assessed using a two-tailed unpaired t-test; "ns" denotes no statistically significant difference. **i** Heatmap of peptide abundances for GAPDH1 and GAPDH2. Rows represent distinct peptides detected and assigned to GAPDH1 or GAPDH2, with peptide sequences listed at right (n=6). **j** Predicted enzyme structures and the interactions between GAPDH1 (FBgn0001091) and GAPDH2 (FBgn0001091) by Alphafold2-multimer. The oxidized methionine and cysteine residues are highlighted. **k** The NAD^+ binding pocket and the surrounding area from GAPDH monomer structure (PDB: 1U8F) is shown. NAD^+ and methionine resides are highlighted in red and purple, respectively. **l** qPCR analysis of dGAPDH1 mRNA Expression. Quantification of dGAPDH1 mRNA expression in muscle tissue from Control (gray) and Muscle>GAPDH1-RNAi (red) male flies using quantitative PCR (qPCR). The thoraces were collected after five days of GAPDH1-RNAi activation. Data are shown as mean ± SD from biological replicates (n = 6). Statistical significance was assessed using a two-tailed unpaired t-test; * $p < 0.05$.

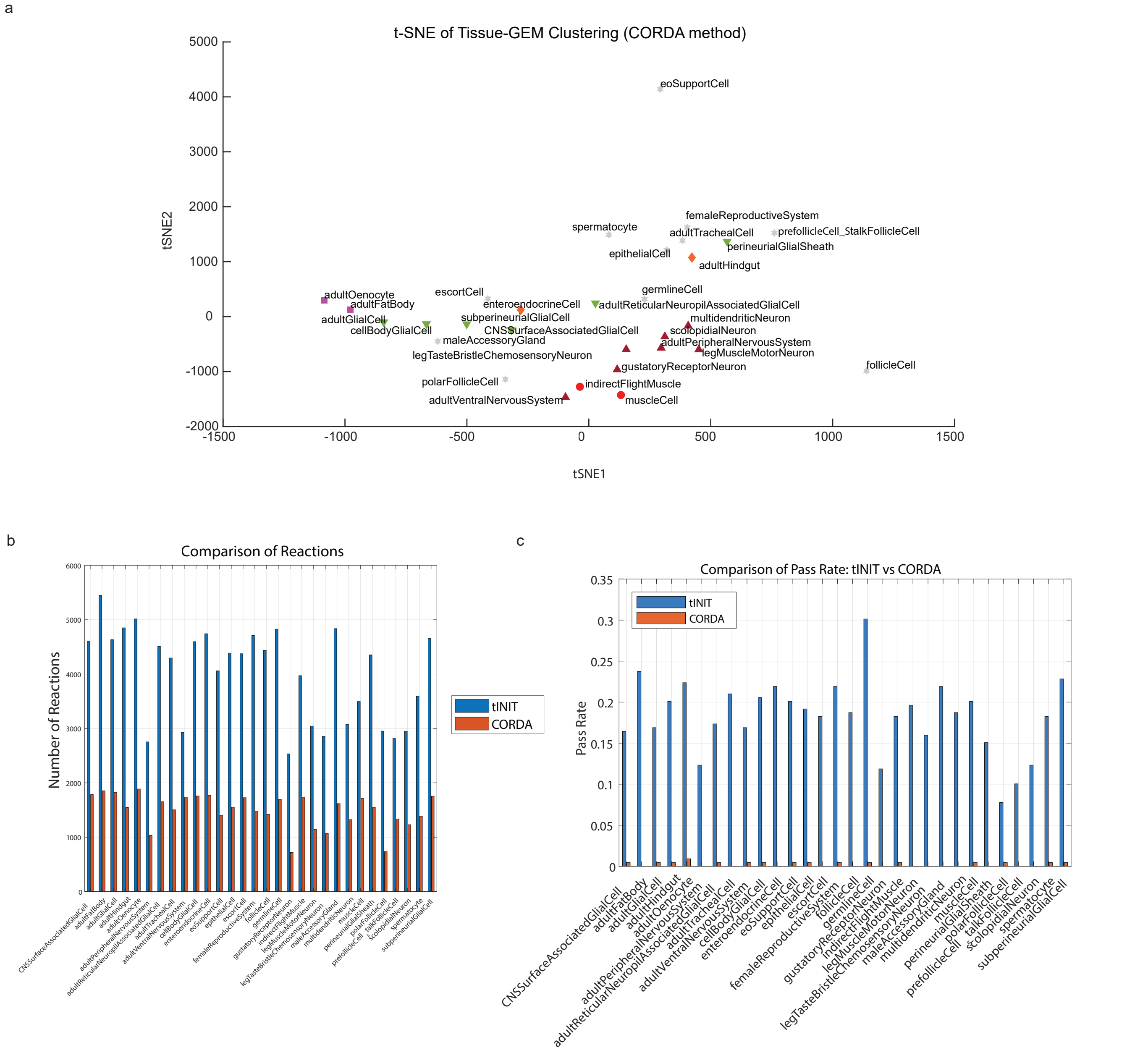
a
t-SNE of Tissue-GEM Clustering (CORDA method)
tSNE1
tSNE2
eoSupportCell
femaleReproductiveSystem
spermatocyte
adultTrachealCell
prefollicleCell_StalkFollicleCell
perineurialGlialSheath
epithelialCell
adultHindgut
adultOenocyte
escortCell
germlineCell
adultFatBody
enteroendocrineCell
adultReticularNeuropilAssociatedGlialCell
subperineurialGlialCell
multidendriticNeuron
adultGlialCell
cellBodyGlialCell
CNSSurfaceAssociatedGlialCell
scolopidialNeuron
maleAccessoryGland
adultPeripheralNervousSystem
legTasteBristleChemosensoryNeuron
legMuscleMotorNeuron
gustatoryReceptorNeuron
follicleCell
polarFollicleCell
indirectFlightMuscle
adultVentralNervousSystem
muscleCell
b
Comparison of Reactions
Number of Reactions
tINIT
CORDA
c
Comparison of Pass Rate: tINIT vs CORDA
Pass Rate
tINIT
CORDA
CNSSurfaceAssociatedGlialCell
adultFatBody
adultGlialCell
adultHindgut
adultOenocyte
adultPeripheralNervousSystem
adultReticularNeuropilAssociatedGlialCell
adultTrachealCell
adultVentralNervousSystem
cellBodyGlialCell
enteroendocrineCell
eoSupportCell
epithelialCell
escortCell
femaleReproductiveSystem
follicleCell
germlineCell
gustatoryReceptorNeuron
indirectFlightMuscle
legMuscleMotorNeuron
legTasteBristleChemosensoryNeuron
maleAccessoryGland
multidendriticNeuron
muscleCell
perineurialGlialSheath
polarFollicleCell
prefollicleCell
stalkFollicleCell
scolopidialNeuron
spermatocyte
subperineurialGlialCell

Supplementary Figure 6. Comparison of metabolic network structures obtained through tINIT and CORDA algorithms

a t-SNE plot comparing the metabolic network structures of tissue-specific GEMs reconstructed by CORDA algorithm. Each point represents a tissue-specific GEM, positioned by t-SNE dimensionality reduction based on network structure similarities. Five major tissue groups were highlighted – muscle (red circles), fat body/oenocyte (purple squares), neuron (dark red upward triangles), glia (green downward triangles), gut (orange diamonds), and others (gray stars). **b** Bar plot compares the number of metabolic reactions included in tissue-specific metabolic models generated using two different reconstruction algorithms: tINIT (blue) and CORDA (orange). **c** Bar plot compares the metabolic task pass rate of metabolic models reconstructed using two different algorithms: tINIT (blue) and CORDA (orange).

Supplementary notes

Supplementary note 1: Pathway overlap between tissue-specific GEMs and experimental metabolomics

The goal of this analysis was to compare enriched pathways identified from tissue-specific GEMs with those from regional metabolomics data, using GEM subsystem annotations as a shared reference framework. The steps are as follows:

First, enriched pathways were determined from each tissue-specific GEM using subsystem coverage analysis (Methods: "Metabolic network structure, subsystem coverage, and metabolic task analysis"). Second, regional metabolomics data were analyzed using KEGG over-representation analysis (ORA) to determine significantly enriched pathways (Methods: *"KEGG over-representation analysis for metabolomics"*). Third, to enable direct comparison, enriched KEGG pathway identified from metabolomics were mapped to GEM subsystem names using a manually curated one-to-one mapping table (**Supplementary Data 2e**). Where no exact mapping existed, the original KEGG pathway names were retained. Subsequent analyses were conducted using the standardized GEM subsystem annotations. Fourth, pathway overlap was quantified using a weighted Jaccard index, which integrates both pathway overlap and enrichment significance (Methods: *"Quantification of pathway overlap using weighted Jaccard index"*).

Supplementary note 2: Flux map representing central carbon metabolism

This note provides additional context for the flux map analysis shown in **Supplementary Figure 3a** and the corresponding data in **Supplementary Data 3c** and **d**. Specifically, it illustrates how central carbon metabolism, particularly tricarboxylic acid (TCA) cycle, is altered in the HSD-muscle-GEM relative to the control model, based on the FVA sampling analysis.

As described in Methods (*Flux variability analysis and flux sampling analysis*), we calculated representative fluxes (e.g., mean, median, mode, minimum, and maximum flux values) across 13 TCA cycle–associated reactions. These included citrate synthase (KDN), aconitase (mAcon1/2), isocitrate dehydrogenase (IDH3, IDH), α-ketoglutarate dehydrogenase (OGDH), succinyl-CoA synthetase (Scsα1/β), succinate dehydrogenase (SDH), fumarase (FUM), malate dehydrogenase (MDH2), malic enzyme (MEN), and associated metabolite transporters (Dic1-mediated citrate, α-ketoglutarate, fumarate, and malate transport) (**Supplementary Data 3c).**

Comparison of individual reaction fluxes revealed decreases in OGDH and SDH by approximately 50% relative to control, and increases in MEN, MDH2, CS, and IDH by approximately 150%. When these 13 reactions were averaged across conditions, the overall TCA cycle flux decreased by about 50% in the HSD-muscle-GEM. Additionally, we confirmed balanced production and consumption rates for malate, citrate, α-ketoglutarate, fumarate in mitochondrial compartment, consistent with the steady-state assumption used in flux analyses (see **Supplementary Data 3d**)

Together, the model-predicted increase in upper-cycle and decrease in lower-cycle reactions indicate that the individual fluxes in TCA cycle is not strictly same in part due to transporter fluxes, leading to differential changes in individual reaction fluxes in HSD-muscle-GEM relative to the control.

Supplementary note 3: Flux comparisons among FBA, FVA-sampling, and pFBA

To evaluate altered reactions beyond those directly constrained in central carbon metabolism, we systematically compared predicted flux profiles from FBA, FVA-sampling, and pFBA analyses (see **Methods**). Each method offers distinct advantages and limitations: FVA-sampling explores the entire feasible solution space, capturing metabolic flexibility but potentially introducing unrealistically high fluxes through internal cycling reactions. In contrast, pFBA provides a single parsimonious solution that minimizes total flux and suppresses internal cycling to better reflect enzyme economy, but it may overlook alternative flux distributions. Standard FBA falls between FVA-sampling and pFBA. Thus, we reasoned comparing differential fluxes from these methods provided a more robust flux analysis framework, especially for identifying perturbed reaction sets.

3a. Total flux evaluation:

To validate the robustness of pFBA, we compared the total flux and internal cycling reactions after performing the FBA, pFBA, and FVA-sampling flux simulations. Comparison of total flux revealed a substantial decrease with pFBA relative to FBA and FVA-sampling (**Supplementary Figure 3d**). Specifically, total flux decreased by approximately 76 % and 85 % relative to FVA-sampling under NSD and HSD conditions, respectively. Moreover, only about 20 % of reactions carried non-zero fluxes in pFBA, whereas about 80 % of reactions carried non-zero fluxes in FVA-sampling (**Supplementary Figure 3e**). The higher total flux and greater number of active reactions observed in FVA-sampling likely result from the inclusion of fluxes through parallel pathways and internal cycles.

3b. NAD(P)-dependent internal cycling reactions evaluation:

Internal cycling reactions can occur in under-constrained metabolic networks when fluxes circulate, often leading to inflated total flux or unrealistic pathway activity. To evaluate such internal cycles, we focused on NAD(P)-dependent reactions as these reactions frequently participate in reversible loops involving oxidoreductases and dehydrogenases.

We first profiled NAD(P)-dependent reactions in the muscle-GEM. To detect potential cycling reactions, we selected reactions whose fluxes (i) exceeded 10% of the maximum flux bounds and (ii) were at least twofold higher than those obtained from pFBA analysis. Applying these criteria revealed several reactions with elevated fluxes in both FBA and FVA-sampling but with markedly reduced or suppressed fluxes in pFBA (**Supplementary Figure 3f**). These included reactions associated with *P5CDh1* in arginine and proline metabolism, *Aldh-III* in the Miscellaneous subsystem, *CG8888* in retinol metabolism, and *CG2767* in pyruvate metabolism.

3c. Flux boundary effects in FVA-sampling:

In FVA-sampling analysis, flux solutions of some of some unbounded reactions may approach to boundary limits, raising the possibility that the default bounds may be too restrictive relative to the actual feasible solution spaces. Especially, in our muscle-GEM, default reaction flux bounds were set at ±1,000 (arbitrary units, given unavailable precise physiological units for *Drosophila* muscle *in vivo*). While constraining reaction bounds to physiologically relevant values is critical for accurate flux predictions, comprehensive experimental data required to fully constrain all reaction bounds in *Drosophila* GEM was lacking, generating the potential boundary effects from the simulation.

To assess this possibility, we tested three boundary conditions: ±1000 (default), ±10000, and ±50000. For each boundary condition, all default reaction bounds were scaled to the new boundary values while maintaining model-specific constraints (e.g., HSD and NSD). After varying the boundary limits, we performed FVA followed by sampling analysis. To determine reactions approaching boundary saturation, we used two complementary criteria: (i) reactions approaching boundaries (max/min saturation), where maximum or minimum sampled flux values approached within 1% of the imposed boundary (e.g., max flux ≥990 for a +1000 boundary), and (ii) reactions with consistently high flux (median saturation), where median flux values approached within 1% of the boundary magnitude. Although this percentage depends on the precise threshold used to define boundary proximity, we applied a stringent 1% cutoff to conservatively identify reactions potentially affected by numerical limits.

This analysis revealed minimal effects of boundary magnitude on saturation patterns. In both NSD and HSD models, reactions approaching upper bound saturation were only about 2 % of all

reactions across boundary conditions, whereas reactions showing lower bound saturation were about 1 % (**Supplementary Figure 3g**). Similarly, across different boundary conditions, median flux criteria also showed that less than 1 % of reactions were within 99 % boundary limits. These results suggested that the default ±1000 bounds, including some unbounded nutrient uptake constraints, were not overly restrictive for the majority of reactions in our metabolic model.
