# Supplementary material for: Modeling tissue-specific Drosophila metabolism identifies high sugar diet-induced metabolic dysregulation in muscle at reaction and pathway levels
Source: Nat Commun. 2026 Jan 19;17:1692. doi: 10.1038/s41467-026-68395-3 (PMC12910071; doi:10.1038/s41467-026-68395-3)
Supplement: Supplementary file 2 — Description of Additional Supplementary Files [file 41467_2026_68395_MOESM2_ESM.pdf]

Description of Additional Supplementary Files

Title: Supplementary Data 1
Description: Supplementary data on tissue-specific GEM reconstruction and related analyses.

Title: Supplementary Data 2
Description: Supplementary data on region-specific metabolomics and pathway enrichment analyses.

Title: Supplementary Data 3
Description: Supplementary data on constraint-based flux analyses and sensitivity analysis.

Title: Supplementary Data 4
Description: Supplementary data on isotopologue abundances, fractional labeling for *in vivo* 13C-glucose tracing experiments.

Title: Supplementary Data 5
Description: Supplementary data on peptide oxidation for muscle samples under NSD and HSD conditions.

Title: Supplementary Data 6
Description: Supplementary data on pathway-level flux analysis and metabolomics under NSD and HSD conditions.
